# Proteomics informed by transcriptomics for a qualitative and quantitative analysis of the sialoproteome of adult *Ornithodoros moubata* ticks

**DOI:** 10.1186/s13071-021-04892-2

**Published:** 2021-08-11

**Authors:** Ana Oleaga, Angel Carnero-Morán, M. Luz Valero, Ricardo Pérez-Sánchez

**Affiliations:** 1 Parasitology Laboratory, Institute of Natural Resources and Agrobiology (IRNASA, CSIC), Salamanca, Spain; 2 Proteomics Section, Central Service for Experimental Research, University of Valencia, Valencia, Spain

**Keywords:** *Ornithodoros moubata*, Saliva, Proteome, LC-MS/MS, SWATH-MS

## Abstract

**Background:**

The argasid tick *Ornithodoros moubata* is the main vector in mainland Africa of African swine fever virus and the spirochete *Borrelia duttoni*, which causes human relapsing fever. The elimination of populations of *O. moubata* would contribute to the prevention and control of these two serious diseases. Anti-tick vaccines are an eco-friendly and sustainable means of eliminating tick populations. Tick saliva forms part of the tick-host interface, and knowledge of its composition is key to the identification and selection of vaccine candidate antigens. The aim of the present work is to increase the body of data on the composition of the saliva proteome of adult *O. moubata* ticks, particularly of females, since in-depth knowledge of the *O. moubata* sialome will allow the identification and selection of novel salivary antigens as targets for tick vaccines.

**Methods:**

We analysed samples of female and male saliva using two different mass spectrometry (MS) approaches: data-dependent acquisition liquid chromatography-tandem MS (LC–MS/MS) and sequential window acquisition of all theoretical fragment ion spectra–MS (SWATH-MS). To maximise the number of proteins identified, a proteomics informed by transcriptomics analysis was applied using the *O. moubata* salivary transcriptomic dataset previously obtained by RNA-Seq.

**Results:**

SWATH-MS proved to be superior to LC–MS/MS for the study of female saliva, since it identified 61.2% more proteins than the latter, the reproducibility of results was enhanced with its use, and it provided a quantitative picture of salivary components. In total, we identified 299 non-redundant proteins in the saliva of *O. moubata*, and quantified the expression of 165 of these in both male and female saliva, among which 13 were significantly overexpressed in females and 40 in males. These results indicate important quantitative differences in the saliva proteome between the sexes.

**Conclusions:**

This work expands our knowledge of the *O. moubata* sialome, particularly that of females, by increasing the number of identified novel salivary proteins, which have different functions at the tick–host feeding interface. This new knowledge taken together with information on the *O. moubata* sialotranscriptome will allow a more rational selection of salivary candidates as antigen targets for tick vaccine development.

**Graphical Abstract:**

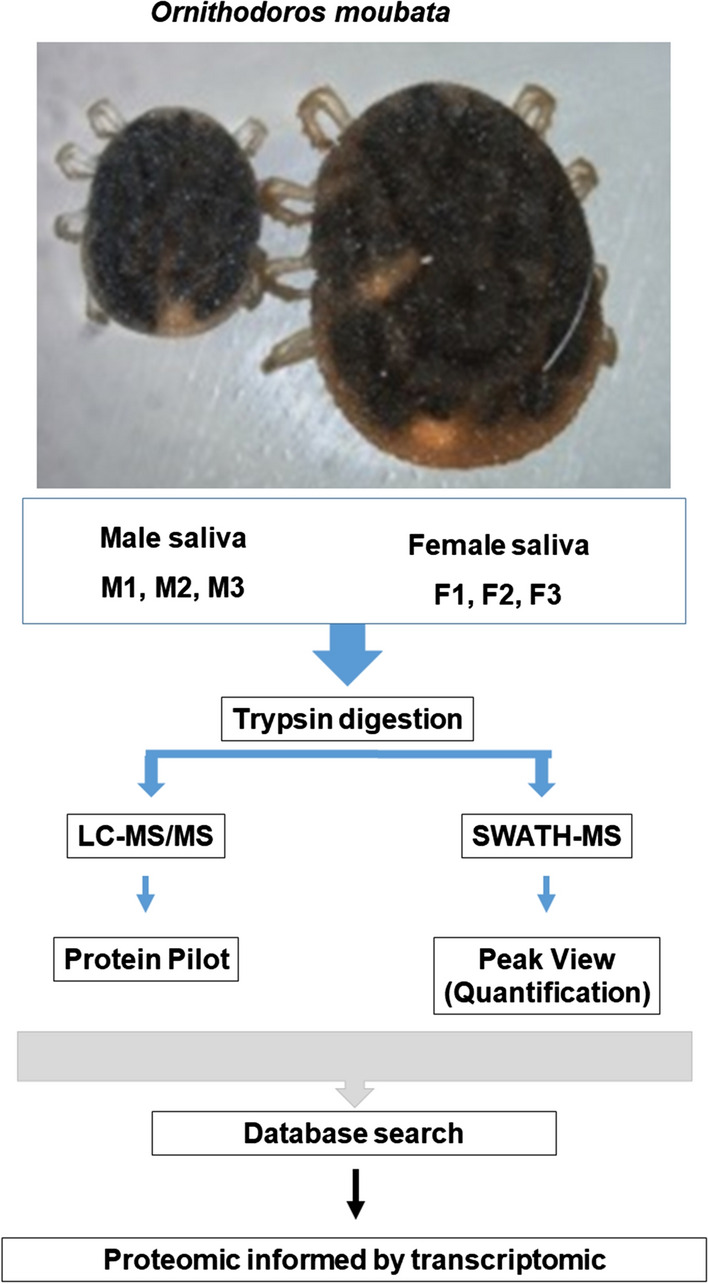

**Supplementary Information:**

The online version contains supplementary material available at 10.1186/s13071-021-04892-2.

## Background

Ticks are considered important blood-sucking arthropods in human and veterinary medicine due to their capacity to transmit a wide variety of infectious agents including viruses, bacteria and protozoans, which can cause severe disease in people and pets as well as in farm and wild animals [1, 2].

The argasid tick *Ornithodoros moubata* has been reported from several countries of southern mainland Africa, including South Africa, Angola, Zambia and Malawi. Unconfirmed historical records of this tick include those from Mozambique, Tanzania, Kenya and Botswana (northern parts); however, as indicated by Bakkes et al. [3], these may prove to be records of different species should they be further studied. *O. moubata* is mainly sylvatic and associated with warthogs and other animals inhabiting burrows, but it is also synanthropic and colonises human dwellings and farm animal housing, particularly that of pigs [4]. In South Africa, Angola, Zambia and Malawi, the medical and veterinary impact of *O. moubata* is significant due to its function as a vector of *Borrelia duttoni*, a spirochete that causes tick-borne human relapsing fever, and of African swine fever virus. Tick-borne human relapsing fever caused by *B. duttoni* is endemic in extensive zones of east Africa, where it affects up to 6.4% of the population and is responsible for perinatal mortality rates as high as 436/1000 [5, 6]. African swine fever virus is an acute febrile haemorrhagic disease of swine, with a lethality rate close to 100%, which limits pig production and causes enormous economic losses in affected countries [7, 8].

In this context, the elimination of domestic and peridomestic *O. moubata* populations would be of great benefit for the prevention and control of these two severe diseases. The main strategy presently used for tick control is the application of chemical acaricides, despite their use being associated with serious drawbacks such as their toxicity, the selection of tick-resistant strains, and contamination of both the environment and animal products [9, 10]. In addition to these drawbacks, chemical agents are ineffective for the elimination of *O. moubata* from human dwellings and animal pens because they do not penetrate holes, cracks and fissures deeply enough to reach the ticks which take refuge in them, as was observed in a study on *Ornithodoros erraticus* in Spain [11].

Anti-tick vaccines have proven to be alternative, eco-friendly and sustainable means of tick control, with clear advantages over chemical agents [12]. Thus, our team initiated the development of a vaccine for the control of *O. moubata* several years ago, with a particular focus on the parasite antigens that form part of the tick-host interface and participate in tick-host interactions, namely salivary and intestinal tick antigens [13].

In the last decade, omics technologies such as next-generation sequencing and high-throughput proteome analysis have been used to explore the sialomes and mialomes of several hard and soft tick species [14–16]. These studies have resulted in the identification of a range of tick molecules involved in the molecular mechanisms underlying tick haematophagy, tick-host interactions and pathogen transmission [14]. We recently applied omics technologies to *O. moubata* to analyse and characterise the transcriptome and proteome of the female tick midgut (mialome) before and after tick feeding [17, 18]. The obtained omics datasets allowed us to select several vaccine candidate antigens, which were produced as recombinant proteins or as synthetic peptides and tested for vaccine efficacy in animal immunisation trials. Some of these candidates proved effective against *Ornithodoros* spp. and thus may potentially be of use in vaccine formulations for tick control [19].

We have recently tackled the in-depth characterisation of *O. moubata* salivary antigens by obtaining and analysing the female salivary transcriptome throughout the trophogonic cycle [20]. The newly obtained data have significantly increased the repertoire of argasid salivary protein-coding sequences available in public databases, and their analysis should facilitate the identification of new antigen candidates for the development of tick vaccines. Moreover, this annotated sialotranscriptome constitutes an invaluable reference database for future studies on the *O. moubata* salivary proteome, and it may be useful to confirm and expand on the data on the salivary proteome of *O. moubata* that were obtained in two former studies [21, 22]. These latter studies are the only ones that have thus far been performed on the *O. moubata* sialoproteome, and their results in terms of protein identification were limited by the scarcity of known tick sequences available at the time.

Oleaga et al. [21] analysed the proteome of the salivary glands of female *O. moubata* by two-dimensional sodium dodecyl sulphate–polyacrylamide gel electrophoresis (SDS-PAGE) and matrix-assisted laser desorption ionisation time-of-flight MS, while the study by Diaz-Martín et al. [22] used liquid chromatography-tandem mass spectrometry (LC–MS/MS) to study the proteome of female and male saliva separately. Diaz-Martín et al. [22] observed noteworthy differences in the proteome composition between the sexes, as well as a high overrepresentation of some lipocalins in female saliva, which hindered the identification of most of the female saliva proteome, which comprises far fewer abundant proteins. Although equalising the saliva samples significantly increased the number of identified proteins, this treatment precluded the quantification of each protein component in the native saliva samples [23].

The specific aim of the present work is to expand the data on the salivary proteome of *O. moubata* adult ticks, particularly of females, by solving the aforementioned drawbacks, as part of the in-depth characterisation and analysis of the *O. moubata* sialome. This information will enable the identification and selection of novel salivary antigens as targets for tick vaccines, which can then be tested in animal immunisation trials. We pay special attention to female ticks because they are a key component of the tick life cycle and vaccines may exert highly deleterious effects on them, for instance, an increase in mortality and a reduction/inhibition of reproductive performance.

In the work presented here, we analysed samples of female and male saliva separately using two different MS approaches: data-dependent acquisition (DDA) LC–MS/MS, and sequential windowed acquisition of all theoretical fragment ion spectra–MS (SWATH-MS). This latter technique is a specific method of data-independent acquisition (DIA), and is an emergent technology that combines deep proteome coverage with quantitative consistency and precision [24].

To maximise the number of protein identifications, we applied a proteomics informed by transcriptomics (PIT) analysis, which used the *O. moubata* salivary transcriptomic dataset previously obtained by RNAseq [20] as a reference database for protein identification. Additionally, besides the analysis of the acquired proteomics data for protein identification and comparative quantification between female and male ticks, a comparative analysis was made of the performance of LC–MS/MS and SWATH-MS and the reproducibility of data obtained with each, which demonstrated greater usefulness and advantages of SWATH-MS over LC–MS/MS for the identification and quantification of the salivary proteins of *O. moubata* females.

## Methods

### Tick specimens

The *O. moubata* specimens used in the present work originate from the Institute of Natural Resources and Agrobiology colony, which was established from specimens donated by Dr Philip Wilkinson (Institute for Animal Health, Pirbright, UK) captured in Malawi. This colony is kept at 28 °C, a relative humidity of 85% and a 12/12-h light/dark photoperiod, and is regularly fed on rabbits.

### Tick saliva collection

Saliva was collected separately from newly moulted 4-month-old female and male ticks after stimulation of secretion with pilocarpine following the protocol described by Baranda et al. [25], with the following modifications. Tick specimens were sequentially washed by immersion and shaking inside a 50-ml disposable centrifuge tube with the following series of 25-ml solutions: tap water, 3% hydrogen peroxide, two washes in distilled water, 70% ethanol and two more washes in distilled water. The ticks were then dried on paper towels and immobilised with double-sided adhesive tape on a glass plate. Each tick was administered 1 µl of 1% pilocarpine hydrochloride (Sigma) in phosphate-buffered saline at pH 7.4 through the genital pore using a 5-µl Hamilton syringe fitted with a 33-gauge, 25-mm-long needle. Shortly after stimulation, the tick started to move the chelicerae and emit small droplets of clear viscous saliva (< 0.5 µl), which were harvested from the tick mouthparts using a micropipette, and deposited on 150 μl of ice-cooled PBS. Saliva was collected from the ticks while perceptible emission continued, usually for 30–40 min after stimulation.

Three replicate biological saliva samples from each sex were prepared, each containing the secretion of 20 female ticks/sample (F1, F2, F3) or 40 male ticks/sample (M1, M2, M3). Saliva samples were centrifuged for 10 min at 12000 *g* and 4 °C, and the supernatants were recovered and stored at − 20 °C. Protein concentration was assessed by measuring the absorbance at 280 nm in a NanoDrop 2000 spectrophotometer (ThermoFisher), and sample reproducibility was checked by SDS-PAGE (Additional file 1: Figure S1).

### Protein digestion and sample preparation

Trypsin digestion and proteomic analyses were carried out at the Central Support Service for Experimental Research at the University of Valencia Proteomics Unit, which is part of the Carlos III Health Institute ProteoRed Proteomics Platform.

Salivary proteins in each female (F1, F2, F3) and male (M1, M2, M3) saliva sample were analysed using an in-solution digestion method. For this, 20 μg of protein per sample was digested with Sequencing Grade Trypsin (Promega) as follows. First, protein samples were reduced using 10 mM dithiothreitol (Sigma) to a final volume of 100 μl and the mixture incubated for 20 min at 60 °C. Then, proteins were alkylated with 5.5 mM iodoacetamide (Sigma) to a final volume of 110 μl and incubated at room temperature for 30 min in the dark. Finally, each sample was digested with 800 ng of trypsin in a final volume of 118 μl and incubated overnight at 37 °C. The digestion was stopped with 12 µL of 10% trifluoroacetic acid (TFA) (Fisher Scientific) in water. The mixtures were dried in a rotatory evaporator and dissolved in a final volume of 40 μl. All the reagents were prepared in 50 mM ammonium bicarbonate (Sigma).

### LC–MS/MS analysis and building a spectral library

The peptides recovered from the in-solution digestion process were analysed in a microESI qQTOF mass spectrometer (6600+ TripleTOF; Sciex) in DDA mode. Briefly, for LC–MS/MS analysis, 5 µl of each digested sample (six samples; three from females and three from males) was individually loaded by using a nanoLC 425 (Eksigent) onto a trap column (3µ C18-CL 120 Ᾰ, 350 μm × 0.5 mm; Eksigent) and desalted with 0.1% TFA at 5 µl/min for 5 min. Then, the peptides were separated using an analytical LC column (3µ C18-CL 120 Ᾰ, 0.075 × 150 mm; Eksigent) equilibrated in 5% acetonitrile (ACN) (Fisher Scientific) 0.1% formic acid (FA) (Fisher Scientific). Peptide elution was carried out with a linear gradient of 7–40% of buffer B in A for 45 min (A, 0.1% FA in water; B, 0.1% FA in ACN) at a flow rate of 300 nl/min. The eluted peptides were ionised in a source type Optiflow applying 3.0 kV to the spray emitted, and the tripleTOF was operated in DDA mode. Full profile MS scans were acquired in the mass range of m/z 350–1400 for 250 ms in positive ion mode. The top 100 most intense ions were selected for fragmentation, and MS/MS scans were acquired in the mass range of m/z 100–1500 for 25 ms in high sensitivity mode. The switch criteria were as follows: charge of 2+ to 4+ , minimum intensity, and 100 counts per second. As described below, the results of these analyses were used in a comparative analysis of the reproducibility and performance of LC–MS/MS in DDA mode and SWATH-MS.

**Fig. 1:**
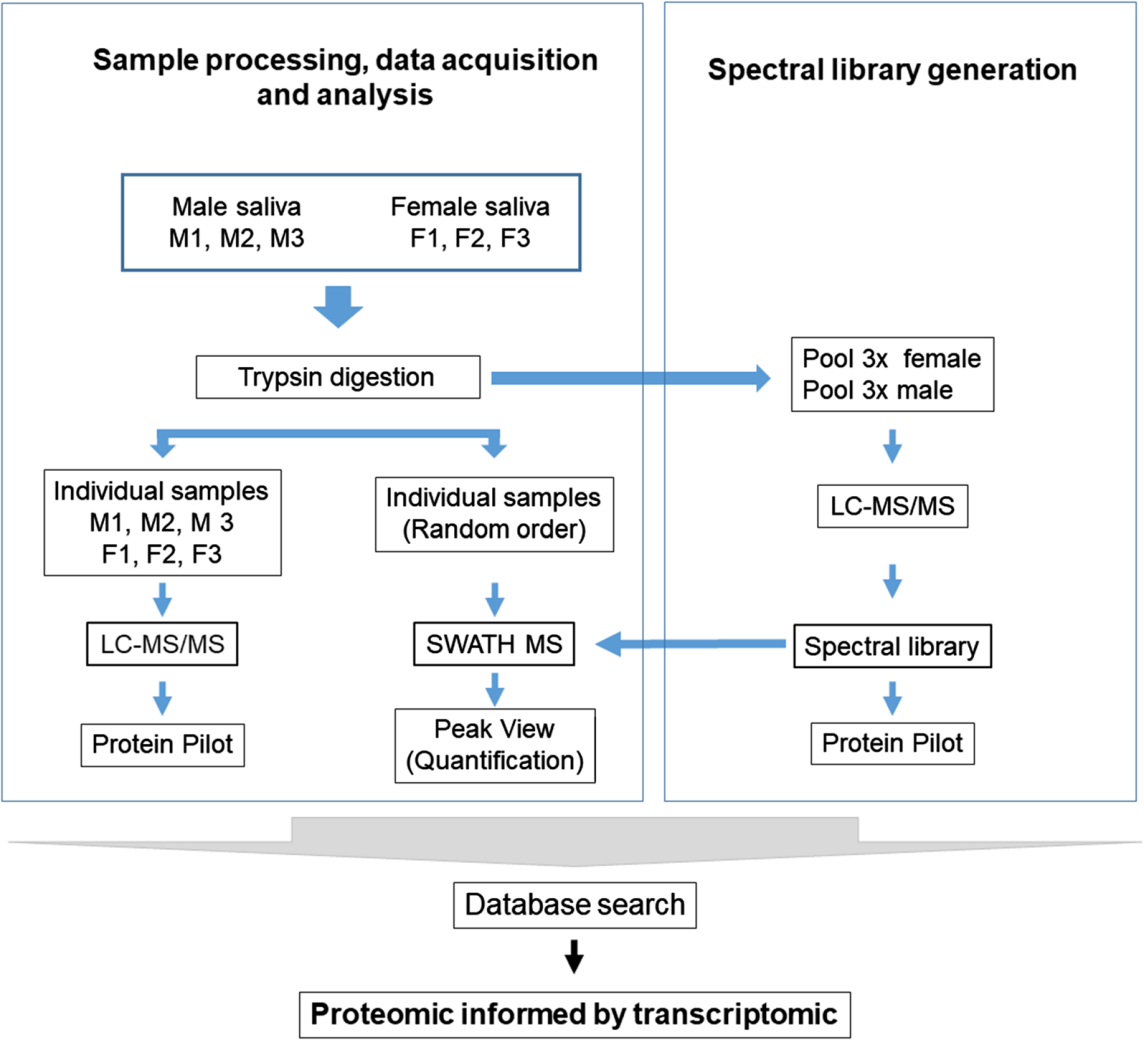
Schematic of the experimental workflows employed in this study. *LC–MS/MS* Liquid chromatography-tandem mass spectrometry, *SWATH-MS* sequential windowed acquisition of all theoretical fragment ion spectra–mass spectrometry

To build a spectral library for SWATH-MS analysis, 2 µl of every digested sample was pooled and 5 µl of the pool was processed and analysed by LC–MS/MS in exactly the same manner as described above for the individual samples. All the spectra obtained were combined and used for the generation of the reference spectral ion library as part of the SWATH-MS analysis. The scheme of the workflow for the spectral library creation, as well as for sample processing, is represented in Fig. 1.

### SWATH–MS analysis

SWATH-MS data for both female and male saliva samples were acquired on the same MS instrument used for LC–MS/MS. The liquid chromatography conditions were as follows. A volume of 5 µl of each digested sample was loaded onto a trap column (12-nm, 3-µ Triart C18, 0.5 × 5.0-mm LC column; YMC) and desalted with 0.1% TFA at 10 µl/min for 5 min. The peptides were then loaded onto an analytical column (3-µm polar C18, 150 × 0.3-mm, Luna OMEGA LC column; Phenomenex) equilibrated with 3% ACN and 0.1% FA. Peptide elution was performed with a linear gradient of 3–35% B in A for 45 min (A, 0.1% FA; B, ACN, 0.1% FA) at a flow rate of 300 nl/min.

The samples were acquired in a random order to avoid bias in the analysis. The samples were ionized in a source type Optiflow 1–50 µL micro, applying 4.5 kV to the spray emitter, and the analysis was carried out in DIA mode. Survey MS1 scans were acquired from 400 to 1250 m/z for 250 ms, and 100 variable windows from 400 to 1250 m/z were acquired throughout the experiment. The total cycle time was 2.79 s. The quadrupole resolution was set to UNIT for the MS2 experiments, and data were acquired from 100 to 1500 m/z for 25 ms in high sensitivity mode.

### Identification and quantification of proteins

After LC–MS/MS, the WIFF data files were processed using ProteinPilot v5.0 search engine (Sciex). The Paragon algorithm [26] (ProteinPilot) was used to search against a recently published FASTA protein database (54,812 sequences) derived from the *O. moubata* sialotranscriptome [20] (BioProject PRJNA667315). Searches were done with trypsin specificity, Cys alkylation, and the search effort set to rapid. To avoid using the same spectral evidence for more than one protein, the identified proteins were grouped based on MS/MS spectra by the ProteinPilot Pro Group Algorithm, regardless of the peptide sequence assigned. The protein within each group that could explain more spectral data with a 95% confidence threshold was depicted as the primary protein of the group.

Among the proteins identified by LC–MS/MS in DDA mode in each saliva sample, only those showing a ProteinPilot unused score above 1.3 (> 95% confidence threshold) and a false discovery rate (FDR) lower than 1% were considered significant and included in the ensuing analyses. After manually inspecting all of the proteins identified by LC–MS/MS, redundant identifications were removed by selecting the proteins with the highest score, and the hits of non-annotated transcripts were removed as well.

The proteins identified by SWATH-MS were quantified using PeakView 2.2 software from normalised label-free quantification intensity data. The generated spectral library was used as a database in Peak View 2.2 software for SWATH analysis, and peaks from SWATH runs were extracted with a peptide confidence threshold of 95% and FDR lower than 1%. It was not set to a minimum number of peptides for identification. Quantitated proteins areas were normalised by the total area sum for differential expression analysis. Where redundant proteins were identified, the hit with the lowest* P*-value was selected as representative, the normalised area values of the redundant proteins were added, and the fold change re-calculated.

### Bioinformatic analyses

For functional annotations of the proteins identified, UniProt identifiers of the proteins were used to extract the gene ontology terms for biological process, molecular function and cellular component, as well as cross-references in the InterPro, Pfam and Panther databases. The identified proteins were then functionally classified according to gene ontology terms and bibliographic information, using as a model the classification applied by Kim et al. [27] in their study on the proteome of *Amblyomma americanum* tick saliva.

### Statistical analysis

The quantitative data obtained by PeakView were analysed using MarkerView (v1.2; Sciex). First, signal peak areas were normalised by the total area sum. MultiExperiment Viewer (http://www.tm4.org/mev.html) was used to identify the proteins differentially expressed in saliva between female and male ticks using Welch’s* t*-test with Bonferroni correction. Salivary proteins for which the adjusted *P*-value was ≤ 0.05 were considered significantly differentially expressed between female and male ticks. For every protein, its quantity in each sex was expressed as the mean signal peak area in the three replicated saliva samples, and the fold change in expression between female and male saliva was calculated as the ratio between the mean protein areas for females *versus* males. The results of the hierarchical clustering analysis of the differentially expressed proteome profile of female and male samples were shown using a heat map after* z*-score normalisation using Euclidean distances.

## Results and discussion

### Spectral library

Spectral libraries are essential for effective post-acquisition processing of SWATH data because they contain spectrometric data for all peptide precursors and their respective ion fragments, which were extracted from prior DDA MS experiments [28]. In the work presented here, we generated a spectral library for *O. moubata* saliva proteins from DDA MS experiments using three female and three male saliva samples. The ProteinPilot report showing the spectrometric, statistical and identification data in the spectral library generated from *O. moubata* saliva proteins is presented in Additional file 2: Dataset S1. This library includes 5,497 spectra associated with 99% confidence thresholds, corresponding to 3734 distinct peptides and 388 protein sequences with a FDR ≤ 1%. After eliminating, from the list of 388 proteins, up to 65 redundant identifications and 64 hits to non-annotated sequences from the *O. moubata* sialotranscriptome database, we obtained a final list of 259 non-redundant salivary proteins (Additional file 3: Table S1). This list includes all the proteins identified in the saliva of both sexes and was used as our reference library for the analysis of SWATH data.

Classification of these 259 proteins showed that the functional groups and families comprising the highest numbers of them were involved in metabolic processes (*n* = 48), or were proteases (*n* = 28), antioxidants (*n* = 20), protease inhibitors (*n* = 17), or proteins with unknown function (*n* = 36) (Table 1). Typically, these families and groups are also the most abundantly represented in the sialomes of the other soft and hard tick species that have been analysed to date [16].

**Table 1:** Number of salivary proteins in the spectral library

Classification (functional groups and families)	Number of proteins
Antioxidant/detoxification	20
Cytoskeletal	14
Extracellular matrix	5
Glycine rich	8
Heme/iron binding	2
Immune-related/defence	8
Lipocalins	15
Metabolism, amino acids	2
Metabolism, carbohydrate	13
Metabolism, energy	14
Metabolism, lipids	11
Metabolism, nucleic acids	8
Nuclear regulation	7
Protease	28
Protease inhibitor	17
Proteasome machinery	6
Protein modification	14
Protein synthesis	1
Regulation	15
Signal transduction	6
Transporters/receptors	8
Transposable element	1
Unknown function	36
Total	259

More details can be found in Additional file 3: Table S1

### Results obtained by LC–MS/MS in DDA mode

**Fig. 2:**
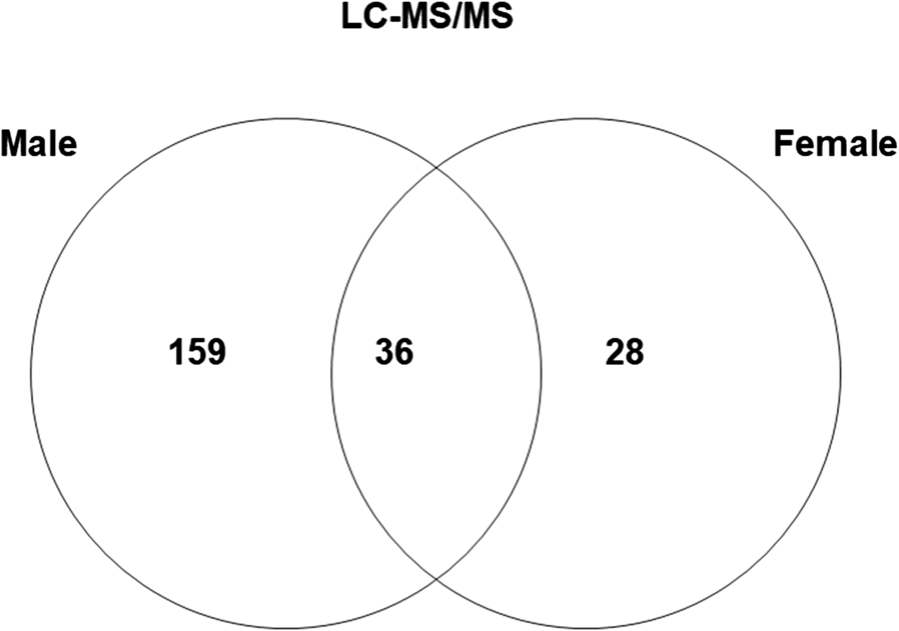
Venn diagram depicting the number and overlap of non-redundant salivary proteins detected by LC–MS/MS

Besides generating the spectral library, we analysed each sample of female and male saliva individually by LC–MS/MS. For simplicity, the lists of protein identifications obtained in the analyses of the individual replicated samples were combined in two unique lists, one for each sex, which were later filtered by eliminating the redundant proteins and the non-annotated hits. Table 2 and Additional file 3: Table S1 show 195 and 64 proteins identified in male and female saliva, respectively. Of these, 36 proteins were shared by both sexes, and 159 and 28 were unique to males and females, respectively (Fig. 2).

**Table 2:** Number of proteins identified in saliva of female and male ticks by liquid chromatography-tandem mass spectrometry (*LC–MS/MS*) and sequential windowed acquisition of all theoretical fragment ion spectra–mass spectrometry (*SWATH-MS*)

Classification	LC–MS/MS		SWATH-MS (male and females)
	Male	Female	
Antioxidant/detoxification	15	3	10
Cytoskeletal	10	1	9
Extracellular matrix	4	2	4
Glycine rich	9	1	8
Heme/iron binding	0	2	1
Immune related/defence	8	2	6
Lipocalins	10	11	11
Metabolism, amino acids	1	1	1
Metabolism, carbohydrates	13	2	8
Metabolism, energy	13	1	12
Metabolism, lipids	10	2	6
Metabolism, nucleic acids	7	3	3
Nuclear regulation	0	0	1
Protease	18	12	19
Protease inhibitor	15	5	9
Proteasome machinery	3	0	2
Protein modification	14	1	12
Protein synthesis	1	0	1
Regulation	11	2	10
Signal transduction	6	0	3
Transporter/ receptors	7	1	5
Unknown function	20	12	24
Total	195	64	165

Some of the differences in protein composition between the saliva of *O. moubata* females and males can also be observed in the different protein band patterns shown by SDS-PAGE (Additional file 1: Figure S1). This had already been described by Díaz-Martin et al. [22], who also confirmed the massive presence of lipocalin proteins in female saliva. A superabundance of lipocalins would have hampered the detection of the less abundant proteins, whose levels most likely would have remained below the limit of detection of the assays. Proof of this was the fact that when the saliva samples were equalised and the “excess” superabundant proteins removed, the number of proteins identified in female saliva increased significantly [22, 29]. At the time of its publication, the work of Díaz-Martín et al. [22] provided interesting novel information on the saliva proteome of males and females, and revealed for the first time differences in the salivary composition of male and females soft ticks. However, that study had one drawback: the method applied to increase the number of identifications, namely, protein equalisation with the ProteoMiner Kit (BioRad), which precluded quantification of the components.

In ixodid ticks, observed differences between males and females in salivary composition are not surprising, since the feeding behaviour and the anatomy and functions of the salivary glands of the sexes differ [30, 31]. However, the differences reported between *O. moubata* males and females in salivary composition were somewhat surprising, since typically soft tick adults, and specifically *O. moubata* adults, do not show anatomical differences in their salivary glands and feed in a similar way. They ingest equivalent amounts of blood relative to their body weight and do so for a similar length of time, about 1 h. This means that both sexes have to overcome the same barriers and host defence responses to complete blood ingestion, and thus are expected to use the same repertoire of salivary proteins for the evasion of host defences [22].

In the present study, the different sets of proteins identified by LC–MS/MS in female and male saliva suggest a qualitative difference in their composition, which agrees with the findings of a previous report [22]. However, it should be noted that the reference database used in the present study for protein identification, i.e. the *O. moubata* sialotranscriptome, was only based on data from female salivary glands. Consequently, it might be assumed that (i) it is most likely that the majority of the proteins identified in the present study, including those found in males only, are also present in female saliva; and (ii) that the differences observed between the sexes are most probably due to quantitative differences in expression rather than to the actual absence/presence of particular proteins in either sex. As discussed below, the results of the SWATH-MS also lend support to this idea.

Accordingly, it can be assumed that some of the qualitative differences observed by Diaz-Martin et al. [22] between the proteomes of male and female saliva, of which only 5.2% of the identified proteins were in common, could be due to quantitative differences.

### Comparison of LC–MS/MS and SWATH-MS analyses

To increase the number of protein identifications in both female and male saliva and quantify the protein expression level we analysed the saliva samples by two methods: LC–MS/MS operated in DDA mode, and the free-label quantitative method SWATH-MS that operates in DIA mode.

MS methods that operate in DDA mode are based on the random selection and fragmentation of a fixed number of peptide precursors, generally the most intense peptide ions. On the other hand, in SWATH-MS capture, all ionised peptides of a given sample that fall within a specified mass range are fragmented in a systematic and unbiased fashion using rather large precursor isolation windows [24]. Several published SWATH studies have demonstrated that SWATH-MS increases the sensitivity and the reproducibility of protein and peptide identification across multiple replicates [32, 33]. Therefore, SWATH-MS might identify and quantify a higher number of proteins expressed simultaneously in *O. moubata* male and female saliva than LC–MS/MS, which in turn allows for a comparison of protein expression levels between the sexes. Accordingly, we first assessed the performance and reproducibility of both methods in the identification of the *O. moubata* salivary proteome by comparing the results obtained by LC–MS/MS and SWATH-MS for the three replicate samples of each sex.

**Fig. 3 a–c:**
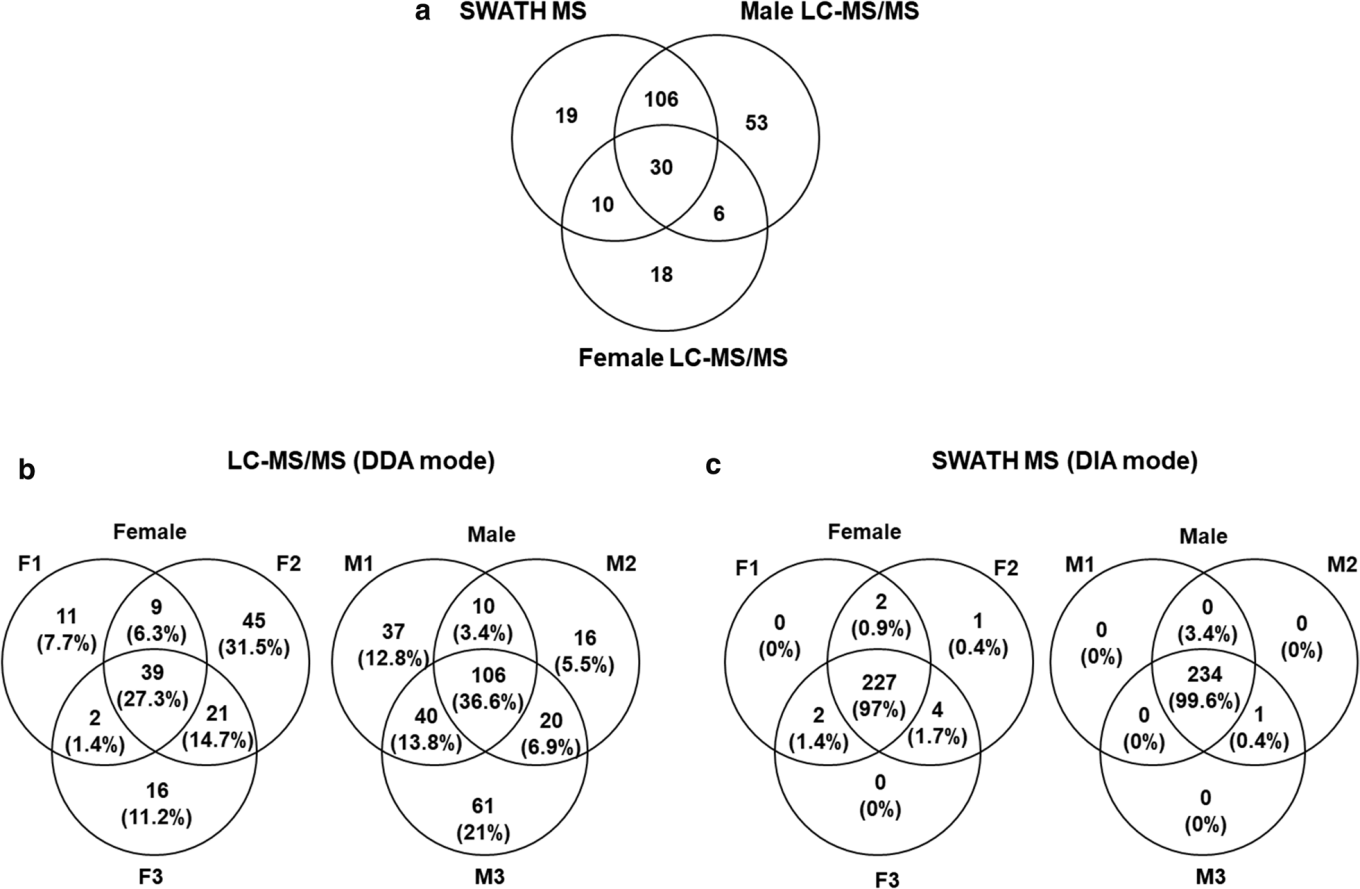
Comparative analysis of the performance of SWATH-MS and LC–MS/MS. **a** Venn diagram depicting number and overlap of salivary proteins detected by LC–MS/MS and SWATH. Venn diagrams depicting the number and overlap of unique proteins detected by **b** LC–MS/MS and **c** SWATH across three biological replicates in female (F1, F2, F3) and male (M1, M2, M3) tick saliva. For abbbreviations, see Fig. 1

Regarding performance, LC–MS/MS analysis of the female samples resulted in the identification of 64 salivary proteins, while SWATH-MS of these same samples identified up to 165 salivary proteins (Table 2; Additional file 3: Table S1). Up to 40 of these proteins were identified by both methods, 24 exclusively by LC–MS/MS and 125 exclusively by SWATH (Fig. 3a). In male saliva, 195 and 165 proteins were identified by LC–MS/MS and SWATH-MS, respectively (Table 2; Additional file 3: Table S1). Up to 136 proteins in males were identified by both methods, 59 solely by LC–MS/MS and 29 solely by SWATH-MS (Fig. 3a).

With a 61.2% increase in the number of proteins identified, our data suggest that in the context of female saliva, SWATH-MS is superior to DDA. However, DDA identified 15.4% more proteins than SWATH-MS in male saliva. These data indicate that the benefit of SWATH over DDA MS, in terms of the number of identified proteins, is unique for the saliva of females, and supports the notion that the performance of these techniques may be dependent on the fluid or the tissue analysed, as has been recently observed [33].

To assess the reproducibility of protein identification by both methods, we compared the data from the three biological replicates of female (F1, F2, F3) and male (M1, M2, M3) saliva. The reproducibility of LC–MS/MS was 27.3% and 36.6% for female and male saliva, respectively (Fig. 3b). On the other hand, reproducibility using SWATH-MS reached almost 100% (97–99%) for both sexes (Fig. 3c). Therefore, SWATH-MS outperformed DDA in the reproducibility of proteins identified across all three technical replicate analyses, and these results are in good agreement with those of previous reports [32]. Additional file 3: Table S1 shows the global results from both MS methods, which jointly identified 299 salivary proteins using the sialotranscriptome of *O. moubata* females as a reference database [20].

### Quantification of the proteins identified in female and male saliva by SWATH-MS

As already noted, SWATH-MS is a DIA method of analysis, and is used to evaluate quantitatively complex samples with high reproducibility [34].

Using this technique, we identified and quantified 165 proteins in the saliva of both female and male ticks, which were later classified into 21 functional groups and families (Table 2; Additional file 4: Table S2). Not unexpectedly, these groups/families coincide with the groups/families more abundantly represented in the *O. moubata* sialotranscriptome [20]. The groups with the highest numbers of proteins were those involved in metabolic processes (*n* = 30) and protein modification (*n* = 12), proteases (*n* = 19), lipocalins (*n* = 11), antioxidants (*n* = 10), regulation (*n* = 10), and of unknown function (*n* = 24).

**Fig. 4 a, b:**
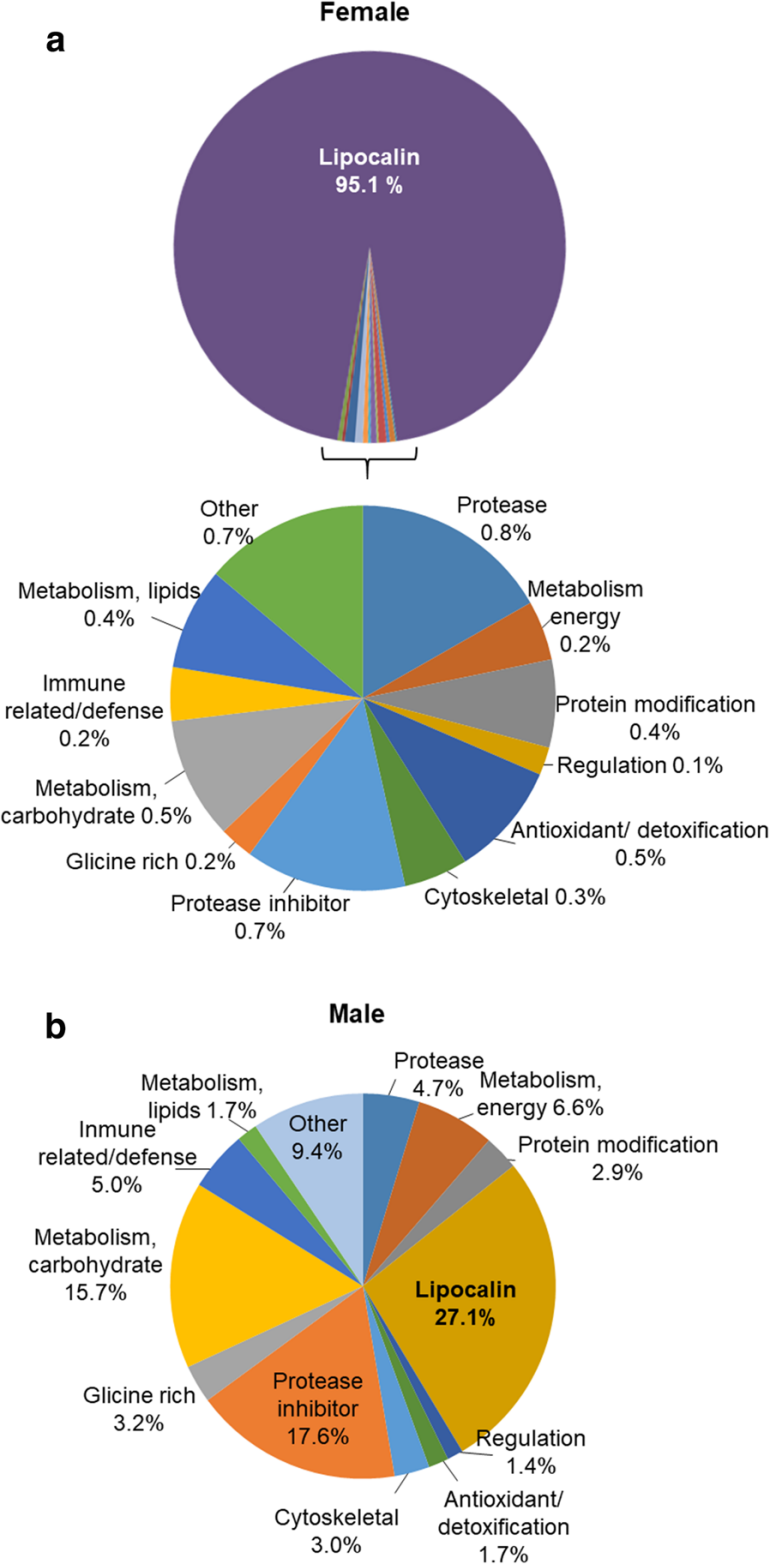
Expression levels of the identified proteins classified into functional groups. The expression level was calculated as the mean spectral signal peak area in female (F1, F2, F3) (**a**) and male (M1, M2, M3) (**b**) samples

Figure 4 represents the expression levels of the protein groups/families in males and females, calculated as the mean spectral signal peak area for female and male saliva (Additional file 4: Table S2). The 24 proteins with unknown function were excluded from the pie charts, and the groups containing five or fewer proteins (proteins involved in metabolism, signal transduction, protein synthesis, extracellular matrix, proteasome machinery and transporters) were merged into one group named “other”.

Female saliva was predominantly composed of lipocalins, which constituted 95.1% of the saliva protein mass (Fig. 4a). This group included 11 proteins, of which moubatin (Q04669) and the so-called salivary lipocalin TSGP1 (F6K8G8) were the most abundant, and respectively accounted for 40.4% and 55.3% of the total mass (Additional file 4: Table S2).

Lipocalins are a large multigene protein family which have dual functions as histamine and serotonin scavengers and as modulators of vertebrate inflammation and immunity [16]. Moubatin belongs to a lipocalin clade that includes proteins that inhibit platelet and neutrophil aggregation by scavenging of thromboxane A2 and proteins that inhibit complement activation by sequestering the C5 component [35]. TSGP1 belongs to the serotonin and histamine-binding group of the soft tick lipocalins [36]. The present results confirm a previous report by Oleaga et al. [21] regarding the great abundance of TSGP1 discovered in the proteome of the salivary glands of *O. moubata*, and support the notion that, in *O. moubata*, TSGP1 is the main scavenger of histamine and serotonin*.* These pro-inflammatory biogenic amines accumulate at the tick-feeding site and need to be efficiently removed for the tick to successfully feed [34]. Regardless of its function, TSGP1 is highly immunogenic and strongly recognised by the serum of pigs bitten by *O. moubata*, which has made it a useful tool for the serological diagnosis of parasitism by *O. moubata* [37]. In fact, a recombinant form of TSGP1 has already been used in several seroepidemiological studies on the exposure of pigs to this tick in Madagascar, Mozambique and Nigeria [38–40].

The remaining non-lipocalin proteins represented only 4.9% of the total protein content of female saliva. Among these, proteases, protease inhibitors, antioxidants, and proteins involved in the metabolism of carbohydrates and lipids were the more abundant groups, representing between 0.8 and 0.4% of the salivary protein content (Fig. 4a). In these groups, the more abundant proteins were a carboxypeptidase (B7QF76), a metalloprotease (Q09JT3), SCO-spondin-like (XP_021004313), enolase (D4P967), aldehyde dehydrogenase (B7QAL5), catalase (A0A2U8T6B2) and phospholipase A2 (M9W8K4) (Additional file 4: Table S2). For several of these proteins, classified as housekeeping proteins, it has been well established that they can also play important extracellular functions at the host-parasite interface, which help ticks to feed [41, 42]. For instance, in *O. moubata*, salivary enolase acts as a pro-fibrinolytic plasminogen activator receptor [43], and salivary secreted phospholipase A2 plays a role as an antagonist ligand of host P-selectin preventing P-selectin-mediated endothelial activation [44].

In quantitative terms, the proteome of male saliva was remarkably different from that of female saliva (Fig. 4b). In male saliva, lipocalins were also the most abundant proteins, although they only accounted for 27.1% of the saliva protein content. TSGP1 (F6K8G8) was the most abundant individual lipocalin, accounting for 65.1% of the protein content of this group (Additional file 4: Table S2). The next most abundant groups were protease inhibitors (17.6%) and proteins involved in carbohydrate metabolism (15.7%) (Fig. 3b), with serpin-2 (Q06B74) (52.3%) and enolase (D4P967) (55.7%) the most abundant respective individual components. Serpin 2 is a well-characterised serine protease inhibitor that inhibits trypsin and thrombin, and interferes with platelet aggregation and blood clotting [45, 46]. If antihaemostatic activity were also experimentally confirmed for serpin-2 from *O. moubata* , it would indicate that it had a similar function to enolase, described above [43], whose pro-fibrinolytic activity helps to maintain host blood fluidity, which helps ticks to feed.

Among the 165 proteins quantified by SWATH-MS in the saliva from males and females, 53 were differentially expressed (*P* < 0.05) between the sexes (Additional file 5: Table S3); 13 proteins were overexpressed in females and 40 were overexpressed in males. The signal peak areas of the differentially expressed proteins in each of the samples analysed are shown using a heat map after* z*-score normalisation, using Euclidean distances. The heat map shows two main clusters comprising the F1–F3 samples and M1–M3 samples, which correspond to the saliva of females and males, respectively (Additional file 6: Figure S2).

**Fig. 5:**
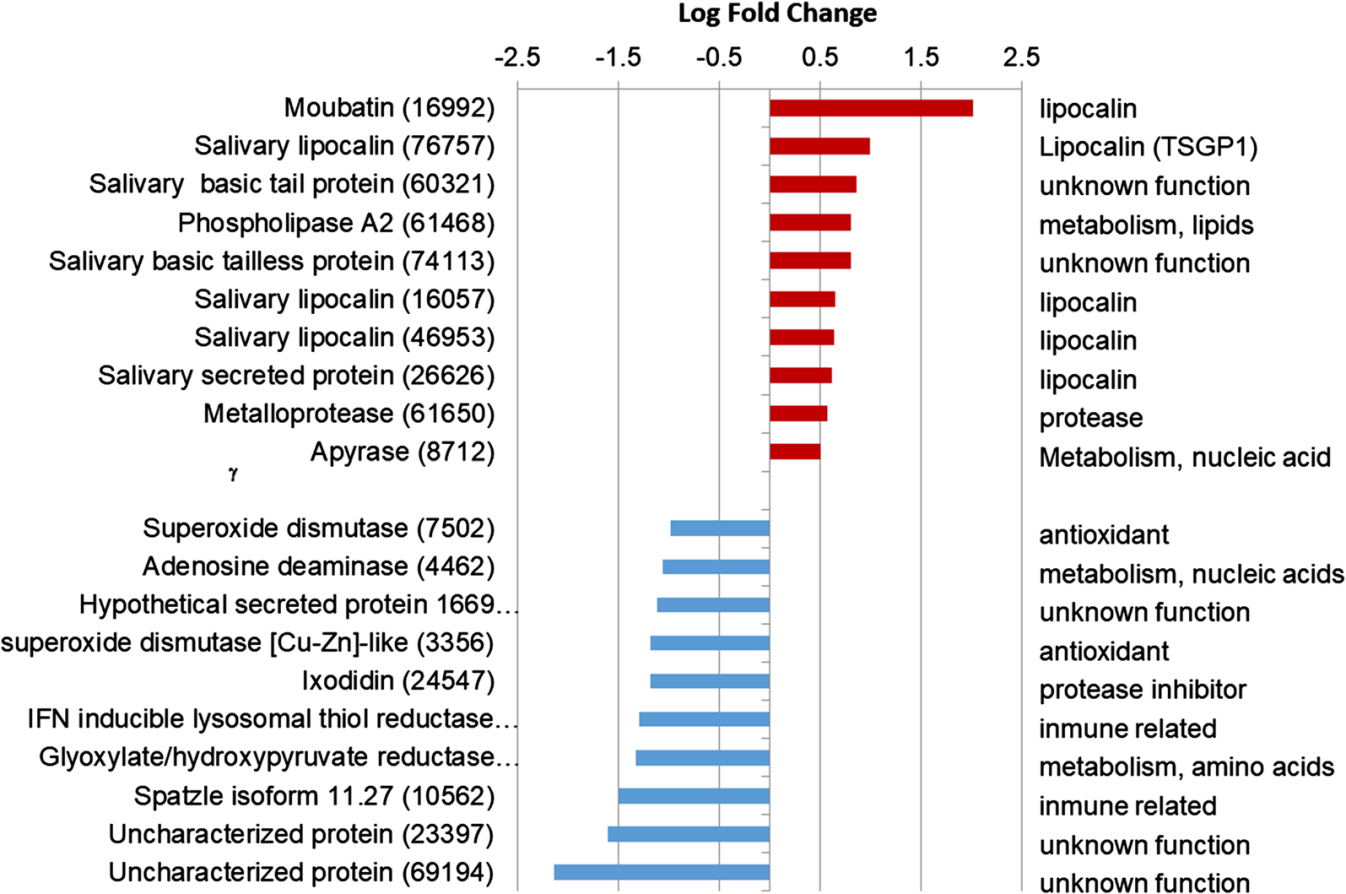
Top ten proteins that are differentially (*P* < 0.05) overexpressed in female saliva (*red*) and male saliva (*blue*). The contig numbers corresponding to proteins are indicated* in parentheses*

Figure 5 shows the top ten proteins that are differentially (*P* < 0.05) overexpressed in the saliva of female and male ticks. As expected according to the results reported above, the top ten overexpressed proteins in females were five lipocalins, including moubatin and TSGP1, phospholipase A2, apyrase, a metalloprotease, a salivary secreted basic tail protein and a salivary basic tailless protein (Additional file 5: Table S3). Apyrase has been identified in the saliva of most haematophagous vectors, including soft and hard ticks. This enzyme hydrolyses ATP and ADP to AMP, and prevents platelet and neutrophil aggregation and thrombus formation, thus facilitating blood feeding [47, 48]. A recombinant form of the salivary apyrase of *O. moubata* induced protective, strong humoral responses in animal vaccine trials that reduced tick feeding and survival [47].

In *O. moubata*, as in ixodid and other argasid tick species, metalloproteases comprise one of the enzyme classes most abundantly represented in the saliva [15, 20, 49, 50]. In this study, LC–MS/MS and SWATH-MS identified 30 proteases; of these, 16 were metalloproteases and five were differentially expressed (*P* < 0.05) by females and males (Additional file 5: Table S3).

Concerning basic tail and tailless proteins, their existence among the overexpressed proteins is not unexpected, as they are protein families abundantly found in the sialotranscriptomes of ixodid and argasid ticks, which suggests that they may play important and specific roles at the tick-host feeding interface [15, 20, 49, 50]. On the other hand, the top ten proteins overexpressed in male saliva were two superoxide dismutases, two metabolic enzymes (adenosine amidase, hydroxypyruvate reductase), two proteins involved in immune mechanisms (gamma-interferon inducible lysosomal thiol reductase, an alternatively spliced isoform of the Spätzle protein), three proteins with unknown function, and ixodidin (Additional file 5: Table S3, Fig. 5). This latter protein is an inhibitor of serine proteinases, and shows antimicrobial activity [51].

Taken together, these results show that at least 165 out of 299 of the salivary proteins identified in the present study are shared by male and female *O. moubata*, which significantly reduces the range of qualitative differences between male and female saliva reported in previous work, where only 5.2% of the identified proteins were found in both sexes [22]. However, remarkable differences were found in the ratios of salivary proteins that males and females secrete, which raises the question of the biological significance of these differences. It can be speculated that this may be related to post-feeding blood processing or attraction and mating [52], but neither our present results nor a revision of the literature offered evidence to support, or rule out, such a notion, or shed light on this.

## Conclusions

Previous studies on the salivary transcriptome of *O. moubata* adults were handicapped by the superabundance of lipocalins, especially in female saliva, that impeded the identification of less abundant proteins. Equalisation of saliva samples increased protein identification but precluded quantification of the protein components of saliva [22, 29]. To overcome these obstacles, we analysed the proteome of the saliva of male and female *O. moubata* via two MS techniques, conventional LC–MS/MS and quantitative label-free SWATH-MS, and carried out a PIT analysis using the recently obtained sialotranscriptome of *O. moubata* females as a reference database for protein identification [20]. SWATH-MS proved superior to LC–MS/MS for the study of female saliva, since it increased by 61.2% the number of identified proteins, enhanced the reproducibility of the results and provided a quantitative picture of the salivary components. Additionally, the PIT analysis demonstrated its usefulness for proteomics studies of *O. moubata*, a non-model organism for which there are no genomic sequences available. PIT is being increasingly and successfully used in proteomics studies of tick saliva, which are fuelled by the increasing number of tick sialotranscriptomes available as a result of the more frequent application of next-generation sequencing techniques to tick salivary glands [17, 31]. We identified 299 non-redundant proteins in *O. moubata* saliva and quantified the expression of 165 of these in both male and female saliva, among which 13 were significantly overexpressed in females and 40 in males. These results provide evidence of important quantitative differences between the sexes in the saliva proteome, and confirm in part results obtained in earlier work using different methodological approaches. These findings expand our knowledge of the *O. moubata* sialome, particularly that of females, by increasing the number of identified novel salivary proteins with different functions at the tick–host feeding interface. The integration of this new information on the *O. moubata* sialotranscriptome will allow a more rational selection of salivary candidates as antigen targets for tick vaccine development and testing in animal immunisation tests. For example, superabundant proteins, such as lipocalins, should not be considered as vaccine targets because their massive presence in saliva would most likely preclude their complete neutralisation by vaccine-induced antibodies. Finally, we believe that the testing of multiantigenic vaccine formulations that include protective intestinal and salivary antigens should be encouraged, since these formulations will target different biological processes and may provide synergic protective effects leading to the development of more effective vaccines for the control of *O. moubata* infestations and pathogen transmission.

## Supplementary Information

**Additional file 1: Figure S1.** Silver–stained 5–20% SDS-PAGE showing saliva (5 µg/lane) of female (F1, F2, F3) and male (M1, M2, M3) *O. moubata* ticks.

**Additional file 2: Dataset S1.** ProteinPilot software report for the spectral library.

**Additional file 3: Table S1.** List of salivary proteins identified in male and females in the spectral library and by LC–MS/MS and SWATH-MS.

**Additional file 4: Table S2.** List of salivary proteins identified by SWATH-MS. Signal peak area, fold change (*FC*) for females *vs* males, logFC and *P-*values are shown.

**Additional file 5: Table S3.** Proteins detected by SWATH-MS that are differentially expressed (*P* < 0.05) in female and male saliva.

**Additional file 6: Figure S2.** Heat map showing levels of differentially expressed proteins (*P* < 0.05) among female and male biological replicates, and hierarchical clustering, showing two main clusters comprising samples F1–F3 and M1–M3 corresponding to the saliva of females and males, respectively.

## Data Availability

The MS proteomics data have been deposited in the ProteomeXchange Consortium database via the PRIDE [53] partner repository with the dataset identifiers PXD025657, PXD025658, PXD025660, PXD025680.

## Abbreviations

ACN: Acetonitrile
DDA: Data-dependent acquisition
DIA: Data-independent acquisition
FA: Formic acid
FDR: False discovery rate
LC–MS/MS: Liquid chromatography-tandem mass spectrometry
PIT: Proteomics informed by transcriptomics
SDS–PAGE: Sodium dodecyl sulphate–polyacrylamide gel electrophoresis
SWATH-MS: Sequential windowed acquisition of all theoretical fragment ion spectra–mass spectrometry
TFA: Trifluoroacetic acid
