# Supplementary figures and images for: Proteomics informed by transcriptomics for a qualitative and quantitative analysis of the sialoproteome of adult Ornithodoros moubata ticks
Source: Parasit Vectors. 2021 Aug 11;14:396. doi: 10.1186/s13071-021-04892-2 (PMC8356541; doi:10.1186/s13071-021-04892-2)

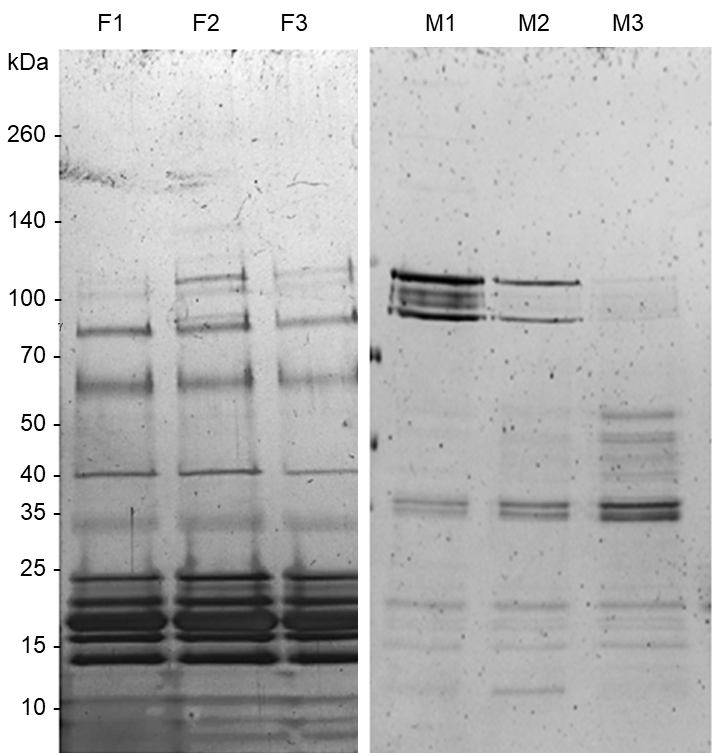

Supplement: Supplementary file 1 — Additional file 1: Figure S1. Silver–stained 5–20% SDS-PAGE showing saliva (5 µg/lane) of female (F1, F2, F3) and male (M1, M2, M3) O. moubata ticks. [file 13071_2021_4892_MOESM1_ESM.tif]

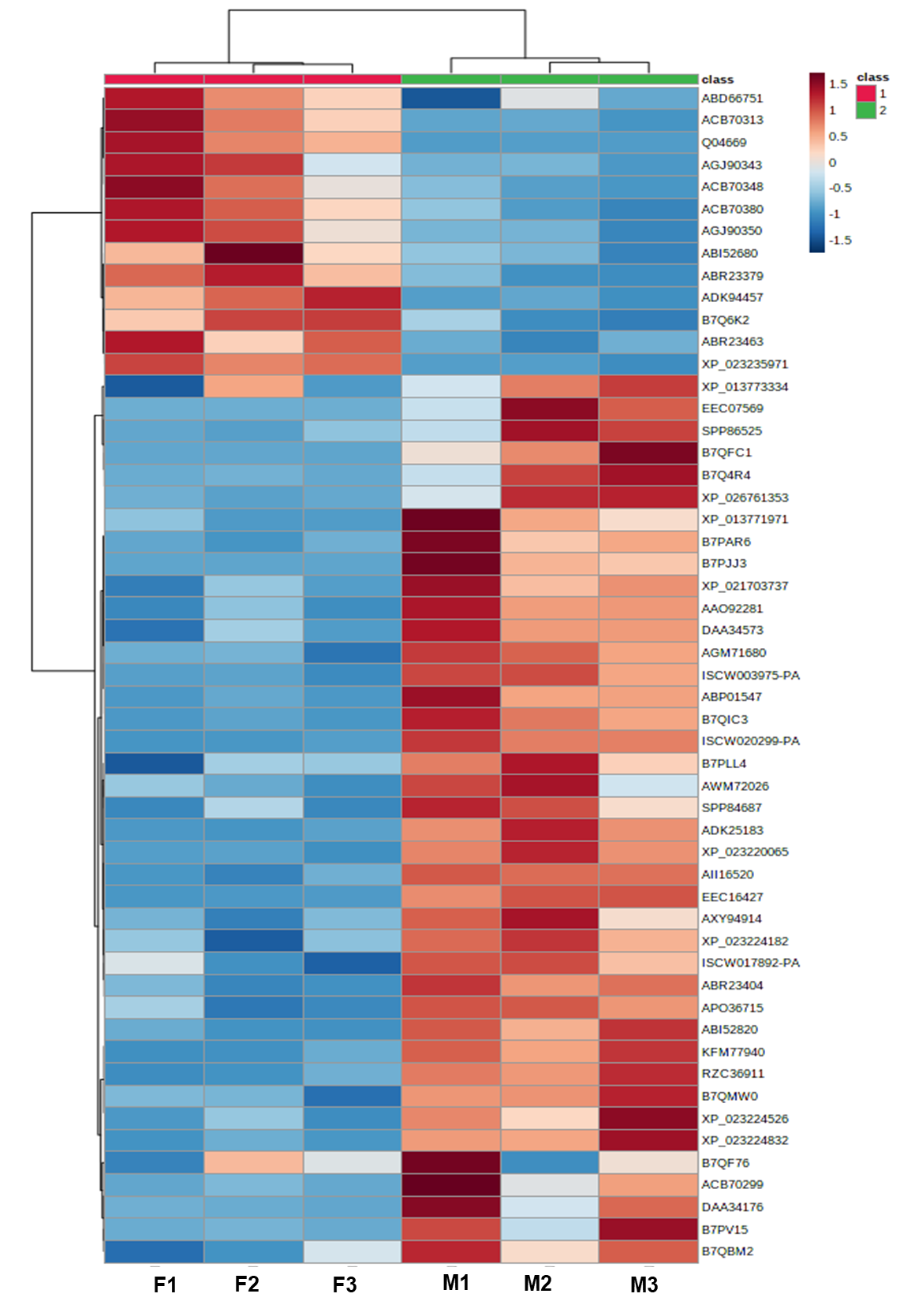

Supplement: Supplementary file 6 — Additional file 6: Figure S2. Heat map showing levels of differentially expressed proteins (P < 0.05) among female and male biological replicates, and hierarchical clustering, showing two main clusters comprising samples F1–F3 and M1–M3 corresponding to the saliva of females and males, respectively. [file 13071_2021_4892_MOESM6_ESM.tif]
