# Supplementary material for: Proteomics informed by transcriptomics for a qualitative and quantitative analysis of the sialoproteome of adult Ornithodoros moubata ticks
Source: Parasit Vectors. 2021 Aug 11;14:396. doi: 10.1186/s13071-021-04892-2 (PMC8356541; doi:10.1186/s13071-021-04892-2)
Supplement: Supplementary file 5 — Additional file 5: Table S3. Proteins detected by SWATH-MS that are differentially expressed (P < 0.05) in female and male saliva. [file 13071_2021_4892_MOESM5_ESM.docx]

Additional file 5: Table S3. Proteins detected by SWATH-MS that are differentially expressed (*P* < 0.05) between female and male saliva. *vs*, versus.

| **Classification** | **Accession** | **Protein name** | **Mean signal peak area (*n* = 3)** | | **Fold Change (female *vs* male)** | ***P*-value** |
| --- | --- | --- | --- | --- | --- | --- |
|  |  |  | **female (*10^4^)** | **male (*10^4^)** |  |  |
| antioxidant/ detoxification | A0A2U8T6B2 | Catalase | 1.11 | 4.83 | 0.23 | 3.87E-02 |
|  | Q09JE3 | Superoxide dismutase [Cu-Zn] | 0.39 | 3.69 | 0.10 | 1.26E-03 |
|  | XP_026761353 | Superoxide dismutase [Cu-Zn] | 0.31 | 4.81 | 0.07 | 2.86E-02 |
|  | A6N9S1 | Thioredoxin peroxidase | 0.51 | 3.24 | 0.16 | 7.70E-04 |
| cytoskeletal | A4UTU3 | Beta-actin | 5.51 | 50.56 | 0.11 | 4.02E-03 |
|  | XP_023220065 | Moesin/ezrin/radixin homolog 1 | 5.63 | 26.56 | 0.21 | 7.10E-04 |
|  | F0JA36 | Profilin | 3.05 | 10.49 | 0.29 | 5.96E-03 |
| extracellular matrix | A0A3G1T1P7 | Mucin-like | 2.31 | 7.13 | 0.32 | 6.90E-03 |
|  | A0A3B0JRB0 | Peritrophin-1 | 1.22 | 4.84 | 0.25 | 1.67E-02 |
| glycine rich | XP_021703737 | Glycine-rich cell wall structural protein 1.8-like | 0.90 | 3.50 | 0.26 | 9.07E-03 |
| immune related/defense | B7Q4R4 | Double sized immunoglobulin G binding protein A | 8.67 | 48.30 | 0.18 | 4.34E-02 |
|  | B7QIC3 | Gamma-interferon inducible lysosomal thiol reductase | 0.19 | 3.71 | 0.05 | 1.27E-03 |
|  | B7QFC1 | Spatzle alternatively spliced isoform 11.27 | 4.22 | 131.89 | 0.03 | 2.34E-02 |
| lipocalin | Q04669 | Moubatin | 4,964.65 | 48.28 | 102.84 | 2.82E-03 |
|  | B2D2A7 | Salivary lipocalin | 84.69 | 19.96 | 4.24 | 2.38E-02 |
|  | B2D2D9 | Salivary lipocalin | 3.98 | 0.90 | 4.40 | 9.64E-03 |
|  | F6K8G8 | Salivary lipocalin (TSGP1) | 6,801.11 | 694.65 | 9.79 | 1.80E-04 |
|  | A6N9Y0 | Salivary secreted protein | 23.36 | 5.77 | 4.05 | 6.85E-03 |
| metabolism | B7PV15 | Glyoxylate/hydroxypyruvate reductase | 2.58 | 55.15 | 0.05 | 4.88E-02 |
|  | B7PLL4 | Fructose-bisphosphatase | 1.04 | 2.04 | 0.51 | 2.18E-02 |
|  | B7PFJ2 | Isocitrate dehydrogenase [NADP] | 0.18 | 1.47 | 0.12 | 5.90E-04 |
|  | XP_013773334 | Lysosomal alpha-glucosidase-like | 8.50 | 12.39 | 0.69 | 4.10E-02 |
|  | XP_023224832 | Acyl-CoA-binding protein-like | 0.20 | 1.33 | 0.15 | 3.74E-03 |
|  | B7QMW0 | Fatty acid-binding protein FABP | 8.94 | 46.22 | 0.19 | 3.48E-03 |
|  | M9W8K4 | Phospholipase A2 | 38.00 | 5.98 | 6.36 | 3.39E-02 |
|  | B7PJJ3 | Adenosine deaminase, putative | 7.85 | 89.73 | 0.09 | 1.57E-02 |
|  | M9WFX8 | Apyrase | 24.37 | 7.79 | 3.13 | 2.96E-03 |
| protease | E0AD92 | Angiotensin-converting enzyme | 8.46 | 44.25 | 0.19 | 1.00E-03 |
|  | A0A087UKQ1 | Carboxypeptidase | 0.58 | 3.45 | 0.17 | 7.00E-04 |
|  | B7QF76 | Carboxypeptidase | 33.37 | 38.82 | 0.86 | 1.41E-02 |
|  | B7QBM2 | Dipeptidyl aminopeptidase III | 0.84 | 1.77 | 0.47 | 2.69E-02 |
|  | A0A482VVE0 | Endothelin-converting enzyme 1 | 2.47 | 17.33 | 0.14 | 7.80E-04 |
|  | Q09JT3 | Metalloprotease | 21.92 | 5.97 | 3.67 | 3.23E-02 |
|  | Q1ZZW9 | Metalloproteinase | 3.20 | 1.12 | 2.85 | 3.82E-02 |
| protease inhibitor | B2D258 | Ixodidin | 0.95 | 14.56 | 0.06 | 4.59E-02 |
| protein modification | A0A1L5L8R3 | Heat shock protein 70-8235 | 0.33 | 1.53 | 0.21 | 2.05E-03 |
|  | XP_023224182 | Heat shock protein 83-like | 3.81 | 11.84 | 0.32 | 7.58E-03 |
|  | B7PAR6 | Heat shock protein, putative | 9.47 | 22.96 | 0.41 | 1.45E-02 |
|  | Q86G69 | Heat shock-related protein | 0.61 | 3.82 | 0.16 | 3.61E-03 |
|  | A0A0A0N845 | Small heat shock protein I | 15.03 | 47.30 | 0.32 | 3.04E-03 |
| protein synthesis | B7Q349 | Elongation factor 1-alpha | 1.34 | 5.84 | 0.23 | 1.70E-04 |
| regulation | XP_013771971 | L-asparaginase-like isoform X1 | 0.11 | 0.78 | 0.14 | 2.86E-02 |
|  | XP_023224526 | Protein/nucleic acid deglycase DJ-1-like | 0.84 | 4.39 | 0.19 | 1.55E-02 |
| signal transduction | A0A076FFP4 | Calmodulin | 0.51 | 2.68 | 0.19 | 7.70E-05 |
| transporter/ receptor | B7PIZ1 | Rab GDP dissociation inhibitor | 2.76 | 8.05 | 0.34 | 1.85E-02 |
| unknown function | XP_023235971 | Ectonucleotide pyrophosphatase/phosphodiesterase family member 1-like | 19.42 | 7.65 | 2.54 | 9.09E-05 |
|  | F0J8E8 | Hypothetical secreted protein 1669 | 10.87 | 141.08 | 0.08 | 2.03E-02 |
|  | A6N9P6 | Salivary basic tailless protein | 3.39 | 0.54 | 6.30 | 3.91E-03 |
|  | B2D272 | Salivary secreted basic tail protein | 21.65 | 3.00 | 7.21 | 8.54E-03 |
|  | A0A3B0JWS0 | Uncharacterized protein | 0.55 | 2.25 | 0.24 | 4.83E-02 |
|  | B7PLU7 | Uncharacterized protein | 0.34 | 46.32 | 0.01 | 4.69E-02 |
|  | B7Q6K2 | Uncharacterized protein | 3.23 | 1.09 | 2.96 | 7.50E-03 |
|  | B7QC55 | Uncharacterized protein | 14.66 | 587.39 | 0.02 | 6.23E-05 |
